# 4D flow CMR detects progressive improvement in ventricular function following cardioversion of atrial fibrillation

**DOI:** 10.1186/1532-429X-18-S1-O89

**Published:** 2016-01-27

**Authors:** Hanna Erixon, Jonatan Eriksson, Ann Bolger, Tino Ebbers, Lars Karlsson, Carl Johan Carlhall

**Affiliations:** 1 Div. of Cardiovascular Medicine, Linköping University, Linköping, Sweden; 2 Div. of Cardiology, University of California San Francisco, San Francisco, CA USA

## Background

Atrial fibrillation (AF) causes impairment of cardiac hemodynamics and substantial cardiovascular morbidity. While AF is difficult to assess with cardiac gated CMR, successful electrical cardioversion of chronic AF is often followed by a transient period of atrial stunning where absence of mechanical atrial contraction, similar to that seen in AF, persists despite reinstitution of sinus rhythm. 4D flow CMR enables assessment of ventricular function according to the volume and kinetic energy of different LV flow components. The volume and end-diastolic kinetic energy (KE) of LV inflow passing directly to ensuing outflow (*Direct flow*) reflect aspects of left atrial-ventricular coupling and have been proven to be markers of LV dysfunction in failing hearts. In this study we hypothesize that left atrial (LA) stunning will contribute to impaired LV function reflected by reduced volume and end-diastolic KE of the LV *Direct flow* component.

## Methods

Eight patients (65 ± 6 years, 1 female) with a history of AF underwent CMR 2-3 hours (scan 1) and 4 weeks (scan 2), respectively, following electrical cardioversion. 4D phase-contrast velocity data and morphological images were acquired at 3T at both scans. A previously validated method was used for the analysis (Eriksson et al., JCMR 2010): The LV endocardium was segmented from short-axis images at end-diastole (ED) and end-systole. Pathlines were emitted from the LV end-diastolic volume (EDV) and traced forward and backward in time until end-systole. Accordingly, the end-diastolic blood volume could be automatically separated into four different functional flow components (Table). The KE was calculated over the cardiac cycle for these flow components based on the volume occupied by each pathline, its velocity, and blood density.

## Results

**Figure 1:**
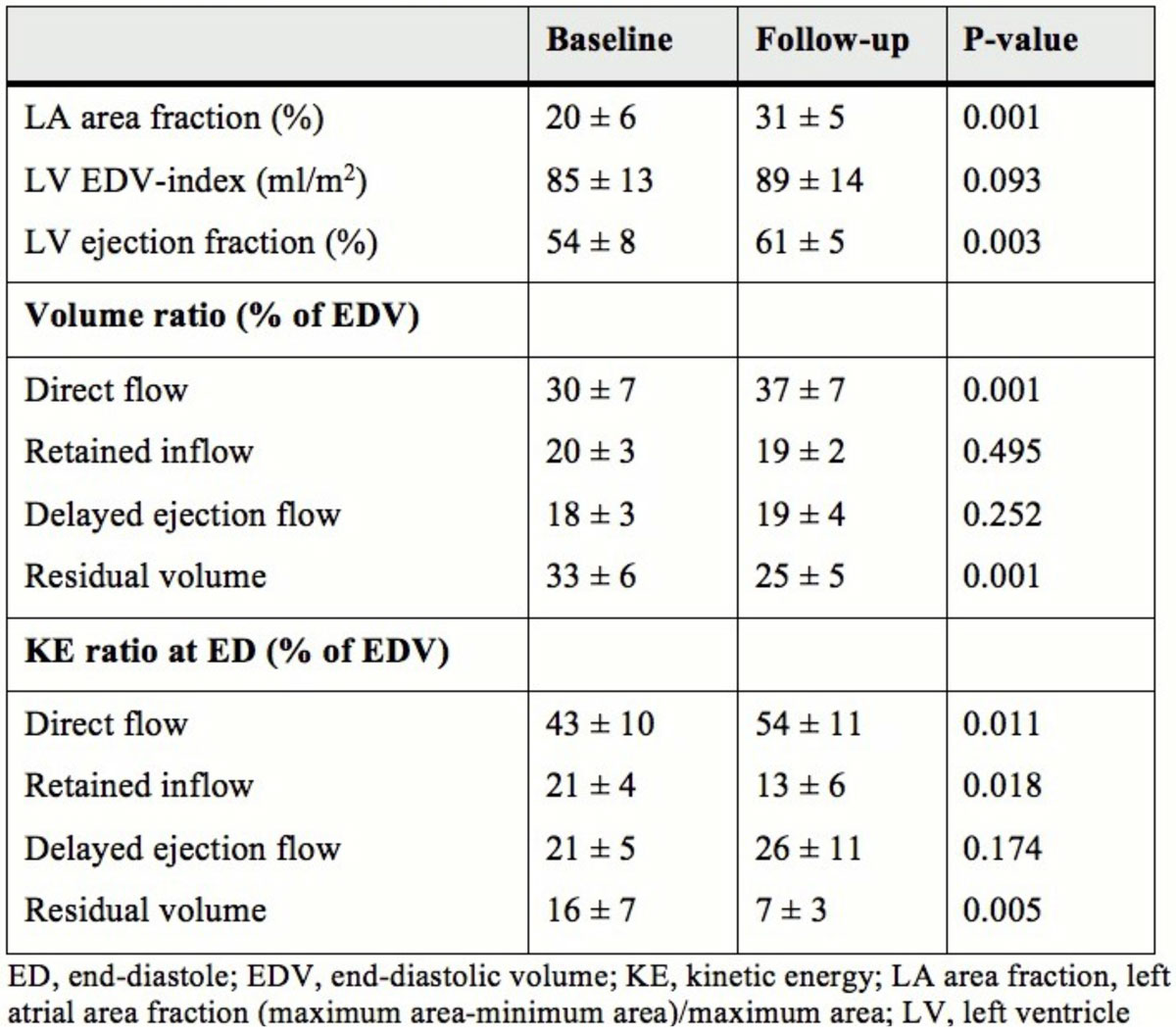
**Left heart dimensions and left ventricular 4D flow measures**.

LA area fraction increased over the follow-up period (P = 0.001), indicating recovery of LA mechanical function (Figure 1). There was no difference in LVEDV-index between the two scans (P = 0.093) whereas LV ejection fraction increased over time (P = 0.003). Regarding 4D flow measures, the *Direct flow*/EDV volume-ratio and KE-ratio at ED increased (P = 0.001 and P = 0.011, respectively), and the *Residual volume*/EDV volume-ratio and KE-ratio at ED decreased (P = 0.001 and P = 0.005, respectively) over time.

## Conclusions

Loss of LA mechanical activity may be a contributor to LV dysfunction and heart failure in AF. During the period of LA stunning following cardioversion, the volume and end-diastolic KE of the *Direct flow* demonstrated impairment of LV function which improved with recovery of LA mechanical function by 4 weeks later. 4D flow specific parameters also showed that the volume and end-diastolic KE of LV residual blood, which may contribute to ventricular inefficiency and stasis of blood, diminished with return of LA mechanical activity. These 4D flow-specific measures may reflect novel aspects of the ventricular benefits of reinstitution of sinus rhythm in the AF patient.

